# Effect of the strigolactone analogs methyl phenlactonoates on spore germination and root colonization of arbuscular mycorrhizal fungi

**DOI:** 10.1016/j.heliyon.2018.e00936

**Published:** 2018-11-22

**Authors:** Boubacar A. Kountche, Mara Novero, Muhammad Jamil, Tadao Asami, Paola Bonfante, Salim Al-Babili

**Affiliations:** a King Abdullah University of Science and Technology (KAUST), BESE Division, The BioActives Lab, Thuwal, 23955-6900, Saudi Arabia; b University of Turin, Life Sciences and Systems Biology Department, Italy; c Graduate School of Agricultural and Life Sciences, The University of Tokyo, 1-1-1 Yayoi, Bunkyo, Tokyo, 113-8657, Japan

**Keywords:** Biochemistry, Plant biology

## Abstract

Strigolactones (SLs), a novel class of plant hormones, are key regulator of plant architecture and mediator of biotic interactions in the rhizosphere. Root-released SLs initiate the establishment of arbuscular mycorrhizal (AM) symbiosis by inducing spore germination and hyphal branching in AM fungi (AMF). However, these compounds also trigger the germination of root parasitic weeds, paving the way for deleterious infestation. Availability of SLs is required for investigating of their functions and also for application in agriculture. However, natural SLs are difficult to synthesize due to their complex structure and cannot be isolated at large scale, as they are released at very low concentrations. Therefore, there is a need for synthetic SL analogs. Recently, we reported on the development of simple SL analogs, methyl phenlactonoates (MPs), which show high SL activity in plants. Here, we investigate the effect of MP1, MP3 and the widely used SL-analog GR24 on AMF spore germination and host root colonization. Our results show that MP1 and MP3 inhibit AMF spore germination, but promote the intra-radical root colonization, both more efficiently than GR24. These results indicate that field application of MP1 and MP3 does not have negative impact on mycorrhizal fungi. In conclusion, our data together with the previously reported simple synthesis, high activity in regulating plant architecture and inducing *Striga* seed germination, demonstrate the utility of MP1 and MP3 as for field application in combating root parasitic weeds by inducing germination in host's absence.

## 1 Introduction

Strigolactones (SLs) are a class of plant hormones and signaling molecules with a wide spectrum of activities. SLs regulate different aspects of plant development [1], which include inhibition of shoot branching [2, 3], determining the architecture of root system [4], formation of adventitious roots, as well as leaf senescence [1]. SLs are also involved in plant's response to biotic and abiotic stress summarized by [5, 6, 7].

SLs were first discovered as stimulants of seed germination of parasitic plants, which are released by host's roots [8]. More recently, SLs were shown to induce spore germination and hyphal branching in arbuscular mycorrhizal fungi (AMF) [9, 10], initiating the establishment of the beneficial AM symbiosis [3, 10, 11, 12, 13]. AMF provide plants with water, phosphorus and other minerals that are absorbed by the wide net of extraradical fungal hyphae. In return, plants supply the heterotrophic partner with organic carbons in the form of sugars and lipids [14] which are required as energy source and for anabolic pathways [15].

Root parasitic plants represent a serious nuisance in agriculture, infesting many crops in warm zones [16]. For instance, *Striga hermonthica*, is one of the most severe threats to food security, since it parasitizes cereals, such as pearl millet, sorghum, rice and maize, causing enormous yield losses, particularly in Sub-Saharan Africa [17, 18, 19]. A single *S. hermonthica* plant can produce up to 200,000 tiny seeds, practically devoid of reserves, that only germinate in close vicinity of host released SLs [20]. Persistent and substantial damage caused by *Striga* spp. to staple food crops of millions of poor rural families led researchers to identify sustainable ways to control *Striga*. The so-called “suicidal germination” – i.e. application of germination stimulants in the absence of host – is a very promising strategy to combat *Striga,* because it offers the possibility to significantly decrease (if not eliminate) the tremendous seed bank residing in the soil. However, natural SLs are not available at the large scale required for this application because of their laborious synthesis and their presence at very low concentrations in root exudates (10^−7^–10^−15^ M) [21, 22, 23]. Therefore, there is a demand for compounds with SL activity, i.e. SL analogs or mimics, which can be deployed as suicidal germinating agents or for other SL-related activities [24, 25, 26].

SLs are carotenoid derivatives characterized by a butenolide ring (D-ring) that is coupled by an enol ether bridge to a tricyclic lactone (ABC-ring) in canonical SLs or to less conserved structures in non-canonical SLs [5, 27]. The synthesis of SLs is initiated by the β-carotene isomerase (Dwarf27, D27) that forms 9-*cis*-β-carotene from the corresponding all-*trans*-isomer [28, 29]. This is followed by a stereospecific oxidative cleavage reaction that is catalyzed by the carotenoid cleavage dioxygenase 7 (CCD7) and leads to β-ionone and a 9-*cis*-configured intermediate [28, 30]. The latter compound is the substrate of CCD8 that catalyzes a simultaneous combination of reactions leading to carlactone [28, 31, 32]. Carlactone is the precursor of canonical and non-canonical SLs, which are formed by the activity of cytochromes P450 (clade 711) and other enzymes [33, 34, 35, 36]. The biological activity of carlactone has led to the development of structurally related analog, nitro-phenlactone [26], and later to a series of compounds termed methyl phenlactonoates (MPs) [37]. MPs showed efficient activities in regulating plant architecture and inducing parasitic seed germination [37]. Moreover, some MPs outperformed GR24, a widely used SL analog with a complex structure, in exerting particular SL functions, such as modulating Arabidopsis root architecture and inhibiting rice-tillering [37]. However, the effect of MPs on AMF, as relevant components of plant microbiota [38], has not been examined so far. Hyphal branching and/or spore germination assays are widely used to assess the activity of SL-analogs on AM fungi [9, 10]. However, these assays only provide insights into their effects during the pre-symbiotic steps of AMF [39], which -during their life cycle- thrive in two niches, the rhizosphere and the root. In this work, we investigated the bioactivity of the two SL analogs MP1 and MP3 on both pre-symbiotic and symbiotic events by considering spore germination and the whole root-colonization process. These findings indicate that both compounds may be used for suicidal germination to combat *Striga* spp without negatively impact the AM symbiotic process.

## 2 Materials and methods

### 2.1 Synthetic strigolactone analogs

**Fig. 1:**
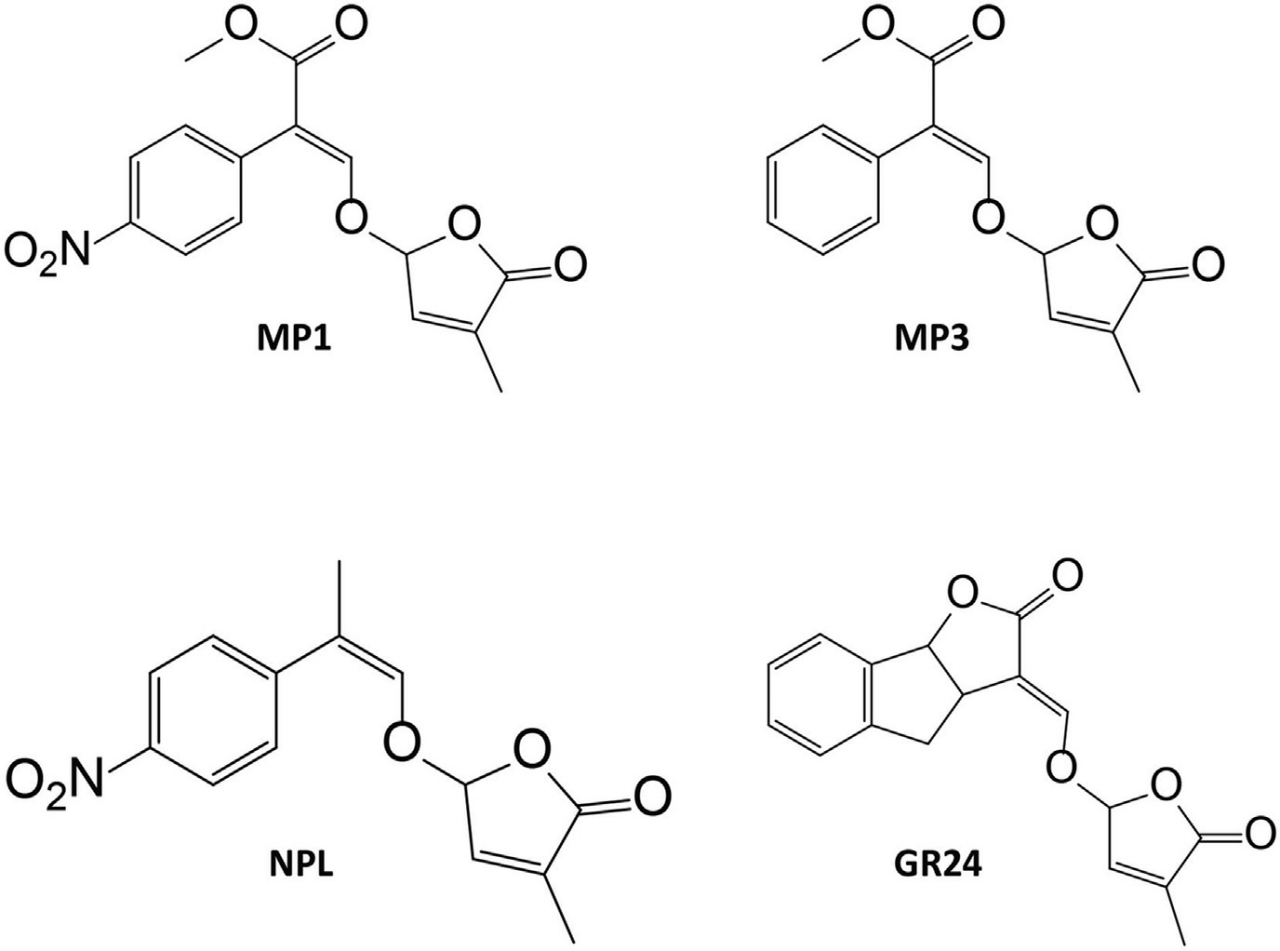
Chemical structure of methyl phenlactonoates, MP1 and MP3 [37], Nitro-phenlactone (NPL) [26] and GR24 [48]. GR24 is racemic.

The SL analogs, MP1 and MP3, were synthetized according to Jamil *et al.* [37]. The standard SL analog (GR24) was kindly provided by Prof. Dr. Binne Zwanenburg (University of Nijmigen; Netherlands) (Fig. 1). All SL analogs used in this study were applied as racemic mixture. Chemicals were first dissolved in acetone to prepare 10 mM stock solution.

### 2.2 Effect of MP1 and MP3 on spore germination of arbuscular mycorrhizal (AM) fungi

To test the effect of MP1 and MP3 on the germination of AMF, spores of *Gigaspora margarita* Becker and Hall (BEG 34) were used. This fungal taxon was selected due to its responsiveness to 5-deoxy-strigol, leading to an increased hyphal branching, mitochondrial density and respiration and the stimulation of the fungal mitotic activity [9, 10, 40]. *G. margarita* spores were obtained by using white clover (*Trifolium repens*) as trap plant: clover plants were inoculated with 100–150 *G. margarita* spores and maintained in pot cultures with quartz sand for at least 3 months. The newly formed spores were then collected by the wet sieving technique [41], stored for 1 week at 4 °C and sterilized with Chloramine T (3% w/v) and Streptomycin sulphate (0.03% w/v) before use. Sterilized spore where then divided in batches of 5 spores each, placed in multiwells plates and germinated for 7 days at 30 °C in the dark. About 1 mL of MP1/MP3 at 10^−7^ and 10^−8^ M were applied per well. The standard SL analog GR24 applied at 10^−7^M and sterile MilliQ water were used as control (mock condition).

### 2.3 Effect of MP1 and MP3 on arbuscular mycorrhizal (AM) colonization

To precisely monitor the impact of MP1 and MP3 application on the interaction with AM fungi, the effect of MP1 and MP3 on the whole colonization process was further assessed. Despite the well-documented roles of SLs in the presymbiotic phase, little is known about the role of SLs in the process of root colonization [42]. To investigate this topic, we used the mycorrhizal system consisting of rice (*Oryza sativa*, cv Nipponbare) and the AM fungus *Funneliformis mosseae*. The partner couple was selected since *i*) rice well responds to SLs and it has been already used to test the two molecules for their effect on plant [37] and *ii*) *F. mosseae* has excellent rice colonization capacities [43], differently from *G. margarita*. Rice seeds were pre-germinated in pots (0.9 L volume) filled with sterile quartz sand and seedlings were placed in plastic Cone-tainer cells (66 mL volume) filled with a mixture 1:10 of *F. mosseae* mixed inoculum purchased from MycAgro Lab (Dijon, France). The mixed inoculum was composed by spores and *F. mosseae* colonized fragments of Sorghum roots. Plants were watered twice a week with 10 mL of tap water plus MP1 or MP3 (10^−7^M and 10^−8^M) or GR24 (10^−7^M) and of Long-Ashton nutrient solution at low phosphate (3.2 μM Na_2_HPO_4_.12H_2_O) plus MP1 or MP3 (10^−7^M and 10^−8^M) or GR24 (10^−7^M). Mock plants were watered only with tap water and Long Ashton nutrient solution. Activity of SL analogs were compared with that of mock plants grown in pots containing only root released SLs. The entire root systems of 3/4 plants (replications) were sampled after respectively 5/7 weeks of co-cultivation. Roots were stained in Cotton Blue 0.1% in lactic acid and the intensity of AM fungal colonization was evaluated according to the method set up by Trouvelot et al. [44]. Effect of the selected MPs on AMF was examined by estimating the frequency (F%) and intensity (M%) of AM colonization, as well as arbuscules abundance (a%) in infected root areas and arbuscules abundance in the whole root system (A%).

### 2.4 Statistical analysis

Data collected from both spore germination and arbuscular mycorrhizal colonization assays were further analyzed using a non-parametric test in order to evaluate the statistical significance between the treatments. Ranked data of both assays were then subjected to Kruskal-Wallis one-way analysis-of-variance-by-ranks test (*P < 0.05*) [45].

## 3 Results

### 3.1 MP1 and MP3 inhibit *G. margarita* spore germination

**Fig. 2:**
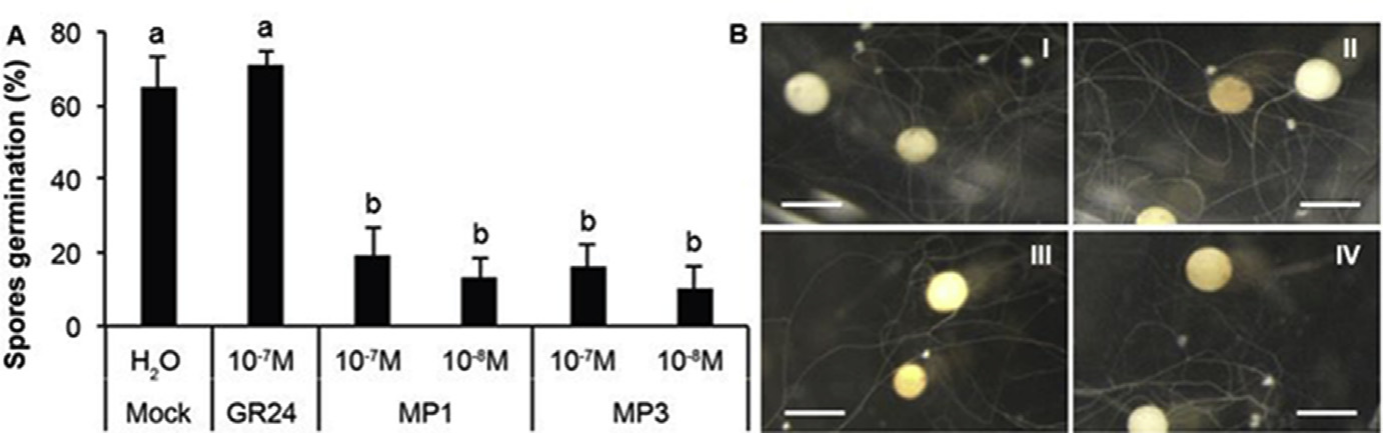
Effect of MP1 and MP3 on spore germination of *G*. *margarita* (A). MP1 and MP3 were applied at 10^−7^ and 10^−8^ M. Concentration for GR24 was 10^−7^ M. Control (Mock) was treated by sterile MilliQ water. Bars represent means of 10 replicates ± SE. Bars with different letters indicate significant difference according to the non-parametric Kruskal-Wallis test (*P < 0.05*). (B) The stereomicroscope pictures illustrate the germinating spores after Mock (I), GR24 10^−7^M (II), MP1 10^−7^M (III) and MP3 10^−7^M (IV) treatments. Bars correspond to 600 μm.

SLs are key player in stimulating spore germination of AM fungi [10]. In this study, we examined the effect of MP1 and MP3 (at 10^−7^ and 10^−8^ M concentrations) on the spores of the AMF *G*. *margarita,* using GR24 (10^−7^ M) and sterile MilliQ water as a control. Fig. 2 shows the highest spore germination rate upon treatment with GR24 followed by water. Treatment with MP1 and MP3 resulted in significantly lower germination rates, irrespective of the concentration (Fig. 2). These results reveal that MP1 and MP3, differently from GR24, exert an inhibitory effect on spore germination of *G*. *margarita*, as seen after 7 days.

### 3.2 MP1 and MP3 promote mycorrhizal colonization

**Fig. 3:**
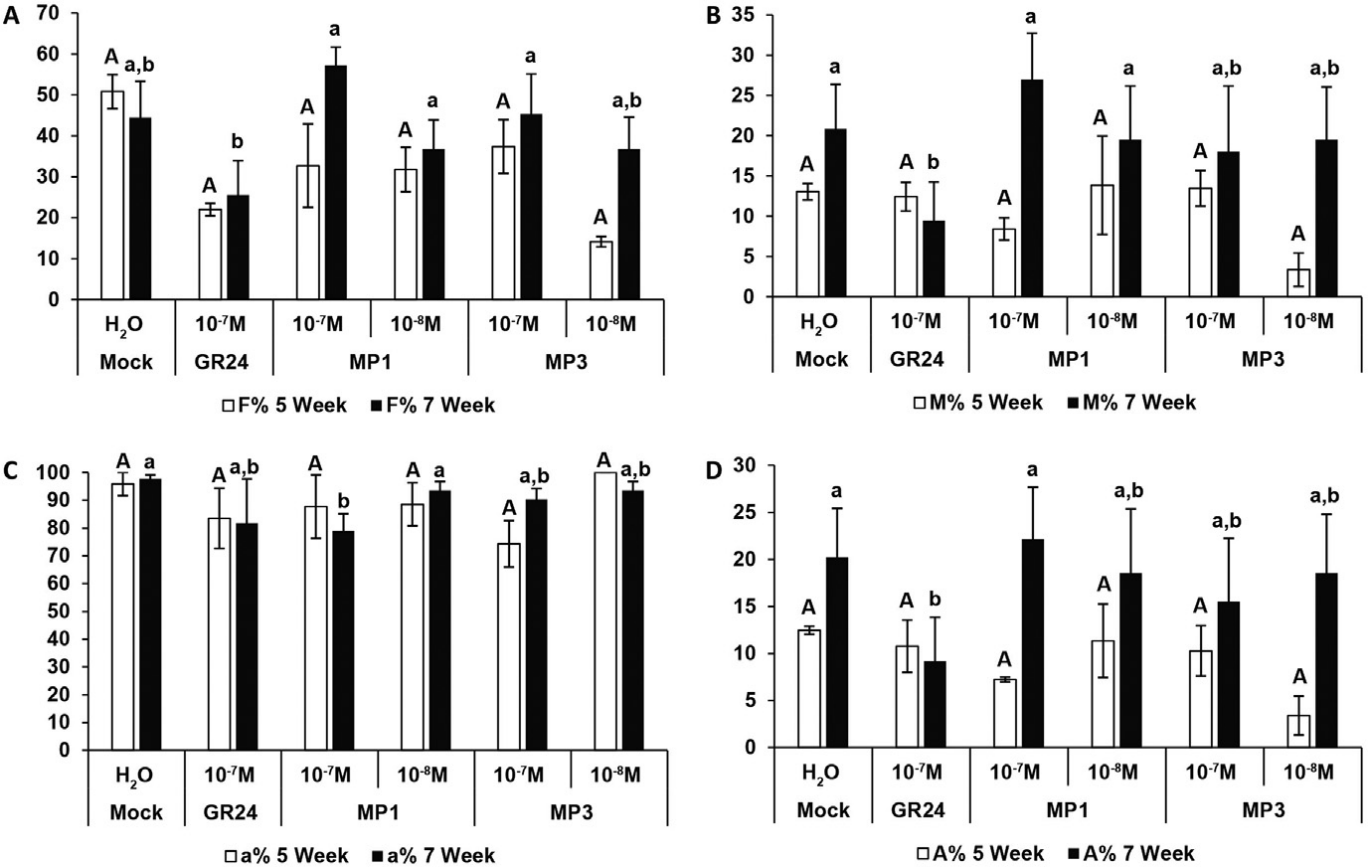
AMF colonization rate in rice roots after treatment with MP1 and MP3. Frequency of mycorrhization in the root system (A), Intensity of the mycorrhizal colonization in the root system (B), arbuscules abundance in infected root (C) and arbuscules abundance in the root system (D) are shown for 5 (white bars) and 7 (black bars) weeks after co-culture. Bars represent mean ± SE (n = 3 at 5 weeks, n = 4 at 7 weeks). Different letters indicate significant difference within each time point, according to the non-parametric Kruskal-Wallis test (*P < 0.05*).

**Fig. 4:**
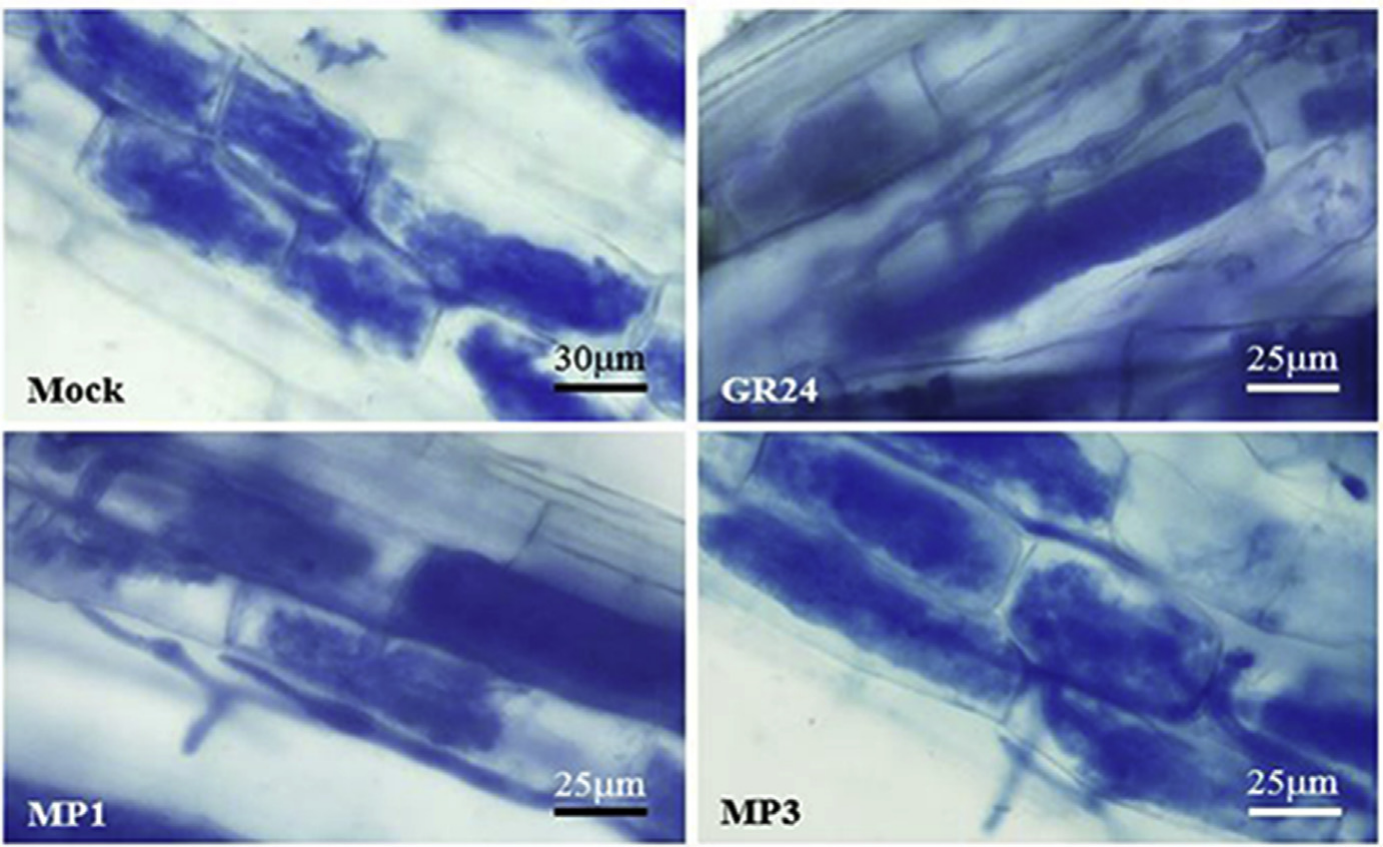
Details of arbuscule morphology in roots stained with cotton blue.

The impact of MP1 and MP3 on the whole colonization process was further investigated to get insights into their effects during symbiotic events. For this purpose, the activity of MP1 and MP3 was compared to that of the standard SL analog (GR24, 10^−7^ M) and to that of the mock treatment, by considering the two time points. After 5 weeks (Fig. 3 white bars), no statistically significant differences emerged among the treatments when four morphological parameters were considered in the roots systems: mycorrhizal frequency (F%), mycorrhizal intensity (M%), arbuscules abundance (A%), and arbuscule development (a%). After 7 weeks (Fig. 3 black bars) the lowest mycorrhizal frequency (F%), mycorrhizal intensity (M%) and arbuscules abundance (A%) in root system were observed upon GR24 treatment (Fig. 3A, B and D). It seems that with this treatment the colonization success is blocked at the values reached after 5 weeks. The arbuscules development (a%) was high, ranging from 79 to 96% in all treatments (Fig. 3C). MP1 and MP3 showed a colonization rate comparable with the mock treatment and when the arbuscule morphology was considered, no differences were detected among the treatments (Fig. 4). The results demonstrate that MP1 and MP3 not only do not have a negative impact on arbuscule development, but also allow the success of mycorrhizal colonization at the later time point (Fig. 3).

## 4 Discussion

The potential of synthetic SL analogs in combating root parasitic weeds by the suicidal germination approach prompted us to develop simple and efficient SL analogs (MPs). Previous experiments showed that these analogs are highly active at relatively low concentrations and that they decompose in the soil in a short time period (Kountche et al., unpublished data, [37]). While the impact of SLs on the pre-symbiotic molecular dialogue between the partners has been largely investigated [46], their role during the colonization process is still to be fully deciphered [42]. Indeed, our findings confirmed the positive effect of GR24 on the pre-symbiotic stage of *G. margarita* (i.e. highest value statistically similar to in water germination), but not for the intra-radical symbiotic phase of *F. mosseae*. In contrast, MP1 and MP3 promoted *F. mosseae* colonization of rice root systems, but showed a rather inhibitory effect on *G. margarita* spore germination [10]. The different AM fungal species were used in the two experiments (germination and colonization), due to their diverse phenotype: one, *F. mosseae*, guarantees an efficient colonization process, thanks to its sporocarps containing many small size spores (30 μm diameter), while the single large (300 μm diameter) spores produced by *G. margarita* were easily handled one by one and followed under the stereomicroscope during their germination process.

Nevertheless, our results allow the conclusion that MP1 and MP3 exert a positive effect on the later stages of the AM colonization process allowing the intra-radical colonization and arbuscule development. This positive effect could eventually counteract the negative one exerted on *G. margarita* germination, and could provide long-term positive effects during field applications. Under natural conditions, AM taxa are present with different dominance: for example a global assessment of AMF diversity performed by considering 1014 individual root samples from vegetation plots from all the continents has demonstrated that *Glomeraceae* to which *F. mosseae* belongs, are dominant when compared to *Gigasporaceae* [47].

Considering their high *Striga* seed germination activity, it has been appreciated that MP1 and MP3 may be potential candidates for application in *Striga* control, to realize the concept of suicidal germination [37]. The impact of MP1 and MP3 on AMF spore germination and AM colonization, make these analogs even more suitable for application as suicidal germination agents, considering that such agents will promote the intra-radical colonization resulting in a rate of AM colonization comparable, or in the case of MP1, higher than that obtained in the mock treatment. The acceptance and implementation of the chemical control by regulatory authorities are based on safety issues, which include avoidance of non-target adverse effects associated with the use of chemical agents. Indeed, these results point out that MP1 and MP3 may facilitate large-scale field application since they are easy to synthetize, efficient and do not negatively impact the successful establishment of the AM symbiosis. They seem, therefore, to successfully satisfy all the parameters required for their practical field application.

## Declarations

### Author contribution statement

Boubacar A. Kountche: Analyzed and interpreted the data; Wrote the paper.

Mara Novero: Performed the experiments; Analyzed and interpreted the data.

Muhammad Jamil: Analyzed and interpreted the data.

Tadao Asami: Contributed reagents, materials, analysis tools or data.

Paola Bonfante: Conceived and designed the experiments; Performed the experiments; Analyzed and interpreted the data; Wrote the paper.

Salim Al-Babili: Conceived and designed the experiments; Analyzed and interpreted the data; Wrote the paper.

### Funding statement

This work was supported by The Bill & Melinda Gates Foundation, Seattle, WA [grant number OPP1136424] given to Prof. Salim Al-Babili and by the King Abdullah University of Science and Technology (KAUST).

### Competing interest statement

The authors declare no conflict of interest.

### Additional information

No additional information is available for this paper.
